# Primary paediatric care models and non-urgent Emergency Department utilization: an area-based cohort study

**DOI:** 10.1186/1471-2296-11-32

**Published:** 2010-05-03

**Authors:** Sara Farchi, Arianna Polo, Francesco Franco, Domenico Di Lallo, Gabriella Guasticchi

**Affiliations:** 1 Public Health Agency, Lazio region, Via di Santa Costanza, 53 00198 Rome, Italy

## Abstract

**Background:**

The aim of this study was to evaluate the association between different primary paediatric practice models (individual, network -affiliated but in separate office-, and group practice) and non urgent utilization of the Emergency Department (ED).

**Methods:**

The data sources were: the 2006 Regional Paediatric Patient files (0-6 years old), the Regional Community-based paediatrician (CBP) file and the 2006 Emergency Information System. We recorded and studied the ED visits of children, excluding planned ED visits, visits for trauma/poisoning and those that were assigned non deferrable/critical triage codes. A multivariate logistic regression was applied to estimate the adjusted odds ratio of an ED visit. The exposure was the type of paediatric practice that served the child: individual, network or group practice. Various characteristics of the child were considered.

**Results:**

The cohort was composed of 293,662 children. In the 2006, 43,347 ED visits occurred (147.6 per 1000). Multivariate logistic models showed lower ED use for group paediatrician patients (OR 0.84; 95%CI 0.73-0.96) and for network paediatrician patients (OR 0.92; 95%CI 0.85-1.00) compared to patients served by an individual practice.

**Conclusions:**

This study shows that there is a weak association between the type of paediatrician primary practice and emergency department use. Our results highlight the necessity to continue to improve the organization of paediatrician primary practice, in order to increase patient access to primary paediatric care.

## Background

The aim of primary paediatric care is to promote children's health through diagnostic, therapeutic and preventive services [1-5]. Primary care should, in addition to promoting child health and managing chronic conditions, address acute conditions by referring patients to specialists and the ED when necessary.

In Italy, as in many countries, the hospital ED is used when there are barriers to access to primary care, especially for children [6]. Determinants of ED use for non urgent conditions are related to difficulties in accessing immediate care by community-based paediatrician [7-10].

The debate on health care reform in several industrialised countries such as the US [11] and Australia [12] focuses on primary care as the sector that coordinates other services and needs to be well-integrated, both internally and with other health- related services, which are crucial in reducing ED access.

In Italy, in order to improve the quality and continuity of care provided by CBPs and in order to reduce inappropriate ED visits, in addition to the traditional primary care model, where each CBP works independently, Italian CBPs have the choice of participating in a network of other paediatric practices while working from their own office, or to work in a group paediatric practice, sharing offices and patient electronic health record systems with other paediatricians. Each CBP in a group or in a network practice is responsible for other CBP's patients from their network/group when they are not available. Network and group practice models are intended to maximize CBP availability and to offer better continuity of care, and have been promoted by the National Health System through an economic incentive.

Similar models operate in other countries where there are groups of Primary Care Paediatricians, such as in Israel, with Health Care Centers [13].

One way to evaluate the effectiveness of different paediatric primary care models is to observe if these models correspond to a reduction of non urgent ED use by their patients [14-17]; for example, in the US the effect of the Medicaid insurance system on hospital and ED use has been evaluated [14,15]. Some other studies [16-19] evaluated the effect of after hours general practitioner cooperative models which ensure close collaboration between primary and emergency care and seems to reduce self referrals at the ED.

To our knowledge no study in Italy has evaluated the effectiveness of these innovative primary care models and how they influence ED use.

This population-based study has been undertaken to evaluate if children treated by CBPs in group paediatric practices or network paediatric practices would have fewer non urgent ED visits than those treated by CBPs in individual practice.

## Methods

The study was carried out in the Lazio region of central Italy; the region has about 5.3 million inhabitants and includes the city of Rome (about 3 million inhabitants). The study period ran from 1^st ^January 2006 to 31^th ^December 2006.

In Italy, almost 100% of children aged 0-6 years are followed by a community-based paediatrician who is employed by the National Health System. All visits to the CBP incur no costs to the patient's family; the paediatrician is reimbursed by the National Heath System on a capitation basis [20]. The CBP provides his patients with: acute ambulatory and home care, coordinates the care of chronically ill patients, consults with subspecialties, provides well-baby checks, writes the requisite certificate for parental absence from work during their children's illness. Outpatient care such as specialist ambulatory services, including diagnostic and treatment procedures, is provided either directly by local health authority services or by accredited public or private facilities that have agreements with local health authorities. CBP referred visits to a specialist, and medical devices incur costs to the patients [21], although chronically ill children or low-income families are exempt from such charges [22]. CBPs also provide primary care to patients outside their catchments area. They provide care during weekday working hours. After hours, on weekends or holidays primary care is provided by non-paediatrician physicians. This service provides night and weekend phone coverage as well as urgent home visits to all patients.

Italy's health care system is regionally-based. The Lazio region in 1999 have formed the regional Public Health Agency responsible for providing technical and scientific support to the regional health departments. The Agency is responsible for the development and analysis of hospital and primary care informative systems and for evaluation of quality of care and epidemiological studies. According to the Italian law n. 196/03 no patient consent is necessary for epidemiological studies aimed at giving population-based measures. The regional Public Health Agency is compliant with the law, and the analysis has been performed on anonymous records.

ED service is a part of the hospital care system, and is provided free of charge. Information on patients, available in the Public Health Agency of Lazio region, were extracted from: 1) the Regional Paediatric Patient file which for each child aged 0-6 years records demographic data, the individual child code, the CBP code and the date when the child was registered with the doctor; 2) the Regional Paediatrician file which contains information about the paediatric practice office address, the type of practice (Individual, Network or Group paediatric practice), the date the practice started and the number of children registered with the doctor; 3) the Emergency Information System (EIS) which collects records from all visits to all 60 EDs in the Lazio region [23]. For each patient it lists: ED name, patient demographic data, arrival information (date, time), triage at arrival in four codes ("red" for urgent, non-deferrable patients, "yellow" for critical but not in immediate danger of death, "green" for less urgent deferrable patients and "white" for patients who should be treated by their general practitioner), up to four diagnoses and up to four therapeutic procedures, according to the ninth revision of the International Classification of Diseases, Clinical Modification (ICD-9-CM) codes, outcome of the visit (discharge, hospitalisation, transfer to another hospital, death, other). "Planned" or "scheduled" emergency visits are those preliminary to an elective hospital admission.

We recorded and studied ED visits of children aged 0-6 years who were patients of the same CBP throughout 2006, who had worked in the same type of practice for at least two years before 2006. All ED visits included in the cohort were identified. We excluded all scheduled ED visits, including hospital admissions, visits for trauma/poisoning ICD-9-CM codes (800-959) and those that were assigned red or yellow triage codes. Finally, we included only green/white triage visits where procedure code was: general physical examination, diagnostic interview, consultation, and evaluation.

The primary outcome variable is the ED visit. The exposures of interest are the three types of paediatric practices: individual, network and group practice.

Potential confounders taken into consideration are: age of the child (categorized into three groups-under 1 year, 1-2 yrs, 3 or more yrs), gender, exemption from payment for specialist outpatient care, place of residence (city of Rome; other areas in the region), as proxy of the distance between CBP office and patient's address, a variable that identifies if the doctor's office was located in the same Local Health Unit of the patient's home (yes, no), and the number of children registered with the doctor (<880 and >880).

A descriptive analysis has been performed to evaluate the characteristics of children included in the cohort and ED visit rates. Adjusted Odds Ratios (OR) and 95% confidence intervals (95% CIs) have been estimated using the logistic regression model. *P*-values have been calculated from the chi-squared test for the distribution across the three types of paediatric practice. A stepwise procedure has been used to include confounding factors into the model. A multilevel approach has been applied to take into account that child patients of the same CBP could have similar socioeconomic characteristics, and type of CBP practice: the micro level is addressed by the child variables, and the macro level is indicated by the CBP information (Model 1).

We reanalysed the association between CBP and ED use, considering only weekdays and daytime ED visits, when the paediatrician's office should be open (model 2); we then excluded weekend ED visits that resulted in a hospital admission (model 3) under the hypothesis that these cases resulted to be severer than the expected and we excluded ED visits in the flu season (November-February) except evening visits that resulted in a hospital admission (model 4).

All the analyses were performed with STATA 8 software.

## Results

The cohort included 293,662 children aged 0-6, patients of 722 CBPs: 67.8% were patients of individual practitioners (489), 20.7% patients of network CBPs (152) and 11.4% of group CBPs (81). In 2006, 151,195 ED visits occurred to the cohort subjects. After excluding 4,871 scheduled/planned ED visits, 34,676 trauma/poisoning visits, 6134 red/yellow triage code visits, and excluding visits where the procedural code did not correspond to physical examination, diagnostic interview, consultation/evaluation, we analyzed 43,347 non urgent visits, corresponding to a rate of 147.6 per 1000 children.

The average number of non urgent ED visits was 1.35, with 82% of infants having had no visits, 13.7% having had one ED visit, and 4% having had two or more visits (range 1-18). Of all ED visits, 11.7% resulted in a hospital admission.

Table 1 shows general characteristics of the study population together with specific ED visit rates and 95% Confidence Intervals.

**Table 1:** Number of paediatric patients by age, types of paediatric practice and ED visits, Lazio, 2006.

	**Patients**		**Individual Paediatric Practice**		**Network Paediatric Practice**		**Group Paediatric Practice**		**ED visits**	**ED visit rate ×1000**	
	**N**.	**%**	**N**.	**%**	**N**.	**%**	**N**.	**%**		**Rate**	***95% CI***
	
**Total**	293,662		199,128		60,912		33,622		43,347	147.6	146.3-148.9
**Age group (yrs)**											
0	43,995	15.0	29,629	14.9	9,244	15.2	5,122	15.2	6,889	156.6	153.2-160.0
1-2	81,755	27.8	55,455	27.8	16,922	27.8	9,378	27.9	17,413	213.0	210.2-215.8
>2	167,912	57.2	114,044	57.3	34,746	57.0	19,122	56.9	19,045	113.4	111.9-115.0
**Gender**											
Males	15,1078	51.5	102,383	51.4	31,360	51.5	17,335	51.6	23,863	158.0	156.0-160.0
Females	142,503	48.5	96,677	48.6	29,543	48.5	16,283	48.4	19,475	136.7	134.8-138.6
**Local Health Unit of residence**											
City of Rome	148,228	50.5	93,196	46.8	36,411	59.8	18,621	55.4	30,022	202.5	200.6-205.2
Other areas in Lazio	145,260	49.5	105,795	53.2	24,477	40.2	14,988	44.6	13,267	91.3	101.3-104.6
**Exempt from payment**											
yes	78,794	26.8	55,198	27.7	14,439	23.7	9,157	27.2	13,358	169.5	166.9-172.2
no	214,868	73.2	143,930	72.3	46,473	76.3	24,465	72.8	29,989	139.6	138.1-141.0

Patient age and gender distribution did not differ by type of paediatric practice, while different types of paediatric practices were observed given a child's residence: 24.6% of children living in the city of Rome were patients of a network CBP, and 12.6% patients of a group CBP, while these percentages were 16.8% and 10.3% among children from the rest of Lazio. Network practitioners had a lower proportion of patients exempt from payment (23.7%). The highest visit rates were shown respectively among children aged 1-2 years (213/1000; 210.2-215.8) and among males (158.0/1000: 156.0-160.0); among children residents of the city of Rome (202.5/1000: 200.5-204.6) and those who are exempt from payment (169.5/1000:166.9-172.2).

Table 2 shows the number of ED visits for the ten most frequent diagnoses that represent 52% of all ED visits. Acute pharyngitis was the most prevalent diagnosis (10%), followed by "symptoms involving the digestive system" (9.2%) and general symptoms (7.9%). Non-significant differences were observed between patients of the three types of practices.

**Table 2:** Ranking of the first ten diagnoses for the ED visit, Lazio 2006

ICD9-CM codes	Description	**N**.	%
462	Acute pharyngitis	4,322	10.0
787	Symptoms involving the digestive system	3,980	9.2
780	General symptoms	3,436	7.9
999	Complications of medical care not classified elsewhere	2,203	4.9
372	Disorders of conjunctiva	2,062	4.6
466	Acute bronchitis and bronchiolitis	1,693	3.8
463	Acute tonsillitis	1,366	3.2
382	Suppurative and unspecified otitis media	1,197	2.8
708	Urticaria	1,192	2.7
381	Non-suppurative otitis media and Eustachian tube disorders	1,163	2.7
	Other diagnoses	20,733	47.8
*Total*		*43,347*	*100.0*

Table 3 shows the crude and adjusted ORs with 95% CIs for emergency department visits for types of practice and confounder factors. Adjusted analysis shows that fewer group CBP patients (OR 0.84; 95%CI 0.73-0.96) and fewer network CBP patients (OR 0.92; 95%CI 0.85-1.00) utilized the ED compared to individual practice. The child characteristics most significantly associated with a higher risk of ED use were: age, under one year (1.42; 95%CI 1.37-1.46) and between 1 and two years (2.18; 95%CI 2.13-2.23); male gender (OR 1.24; 95%CI 1.21-1.27); residence in the city of Rome (OR 2.57; 95%CI 2.41-2.73); children of families exempt from payment of outpatient ambulatory care (OR 1.54; 95%CI 1.50-1.58); CBP in a different Local health Unit than the child's residence (OR 1.37; 95%CI 1.14-1.65); for children treated by a CBP with fewer than 880 patients registered (OR 0.80; 95%CI 0.72-0.89).

**Table 3:** Association between type of practice and ED visit, unadjusted and adjusted by main confounders, Lazio 2006.

	ED visit					
	yes	no	OR	95% CI	OR adj*	95% CI
**Type of Practice**						
Network Paediatric Practice	9,780	41,434	1.12	1.09 - 1.15	0.92	0.85 - 1.00
Group Paediatric Practice	5,066	23,069	1.04	1.01 - 1.08	0.84	0.73 - 0.96
Individual Paediatric Practice	*28,501*	*134,998*	*1.00*		*1.00*	
**Gender**						
Male	23,863	99,615	1.22	1.20 - 1.25	1.24	1.21 - 1.27
Female	*19,475*	*99,830*	*1.00*		*1.00*	
**Age group (yrs)**						
0	6,889	29,378	1.49	1.44 - 1.53	1.42	1.37 - 1.46
1-2	17,413	49,443	2.23	2.18 - 2.28	2.18	2.13 - 2.23
*>3*	*19,045*	*120,680*	*1.00*		*1.00*	
**Exempt from payment patient**						
Yes	13,358	49,452	1.35	1.32 - 1.38	1.54	1.50 - 1.58
*No*	*29,989*	*150,049*	*1.00*		*1.00*	
**Local Health Unit of residence**						
Rome	30,022	98,857	2.30	2.25 - 2.35	2.57	2.41 - 2.73
Other areas in Lazio	*13,267*	*100,548*	*1.00*		*1.00*	
**CBP in the Local Health unit of child's residence**						
Yes	153	718	0.98	0.82 - 1.17	1.37	1.14 - 1.65
*No*	*43,136*	*198,687*	*1.00*			
**N. children patients of the CBP**						
<880	16,761	71,479	1.13	1.11 - 1.15	0.80	0.72 - 0.89
*>881*	*26,586*	*128,022*	*1.00*		*1.00*	

*Adjusted ORs were estimated with multivariate multilevel logistic regression models using all the variables presented in the table. Child variables constitutes the micro level, while CBP information are the macro level

To address the possibility that the relationship between type of practice and ED use was different according to the day of the week, the hour, the season and the outcome of the ED visit, the analysis was also run after excluding different subgroups of the study population (table 4). The results of the other three models were very similar to that of the initial model (model 1) for group CBPs while became not significant for network CBPs.

**Table 4:** Sensitivity analysis according to different criteria of selection^§ ^of ED visit, Lazio 2006

*Model 1 (all subjects)*			
**Type of Practice**	Adjusted OR*	**I.C. adj 95%**	
Network	0.92	0.85	1.01
Group	0.84	0.73	0.96
Individual	1,00		

***Model 2 (excl. ED visits on Saturday and Sunday night (8 pm-8 am)***			

**Type of Practice**	Adjusted OR*	**I.C. adj 95%**	
Network	1.00	0.91	1.08
Group	0.86	0.76	0.97
Individual	1,00		

***Model 3 (excl. ED visits on Saturday and Sunday which outcome was an hospital admission)***			

**Type of Practice**	Adjusted OR*	**I.C. adj 95%**	
Network	0.95	0.87	1.04
Group	0.95	0.85	1.05
Individual	1,00		

***Model 4 (incl. November-February ED visits which outcome was not hospital admission occurred during daytime (8.am-8pm)***			

**Type of Practice**	Adjusted OR*	**I.C. adj 95%**	
Network	1.01	0.89	1.15
Group	0.85	0.72	1.01
Individual	1,00		

*Adjusted ORs were estimated with multivariate multilevel logistic regression models using all the variables presented in the table. Child variables constitutes the micro level, while CBP information are the macro level

## Discussion

This study on non urgent ED visits by children in various types of CBP practice reveals that ED use is not strongly influenced by different primary care models. Nonetheless, a significant lower use of ED in children treated by group paediatricians than those from individual or network paediatrician practices has been observed, after having adjusted for some confounding factors.

Group paediatrician practice is the only form of paediatric primary care that gives children the opportunity to be seen by different paediatricians in the same office. This form of care ensures the presence of at least one CBP every workday. It is possible that group paediatricians working in the same office are able to avoid ED visits for their patients either due to their greater availability or because parents perceive a better "continuity of care" provided by different CBPs working together in the same office. A study in the UK [24] demonstrates that there is a need for providing better education to patients about innovative primary care models, such as after-hours services. It is possible that in our region these innovative forms of paediatric practices have been poorly communicated to patients.

There are few studies which have estimated the impact of different primary care organization models on ED use. Some studies evaluating the effect of innovative primary care models (i.e. longer opening hours) found a minimum impact on diminishing the use of the hospital ED [18]. The poor effect of these models on ED utilization has been explained in different ways: a loss of continuity in the medical care when provided by substitute doctors with no previous relationship with the patient; the perception of immediate care in the ED [25]; the perception of high level of technology and competence in the ED compared to in the CBP office.

On the other hand, a US study demonstrated that a high-quality family-centred primary care model is associated with fewer non-urgent ED visits by children under two years of age [25].

Other factors are associated with ED access for non-urgent conditions, especially among children. A recent study, aimed at providing a comprehensive understanding of motivations of parents of children attending a ED for non urgent conditions, found that the main reasons were the perceiving of a severe condition, or and the high expertise level of the ED doctors [26]. Other important determinants of ED access is the distance between residence and the ED [27]; in our study we couldn't calculate the distance but we used whether the child's residence was located in the same geographical area as the ED as a proxy: it clearly shows that children living near the ED use its services more frequently; moreover, we found that children living in the urban area in Rome, where about 40% of the EDs are located, are twice as likely to visit the ED compared to children living in other areas. Another important determinant of ED use is the socio-cultural level of parents, the lower the socioeconomic level, the more frequent are ED visits, and the more severe are the conditions [28]. A study conducted in Scotland demonstrates that the ED utilization rate for non-urgent conditions is about two times higher in deprived children than in privileged groups [29]. Our study does not permit to analyse non-urgent ED visit rates by socio-economic deprivation factors, but we found that children from families exempt from payment for specialist outpatient care, comparable to receiving public assistance, have higher risk of ED visits. Other determinants of ED use are the age and gender of the children; children aged 1-2 and males are more likely to visit the ED. It could be that infants under one year of age have lower risk of ED visits compared to children 1-2 years old because parents are often in close contact with paediatricians, with fixed appointments and routine visits. It is also possible that infants under one year accessed to the ED as urgent patients. Finally, we found that children assisted by a CBP with fewer than 880 patients are more likely to visit to ED. This finding could be related to a greater availability of CBPs with a large number of patients.

Results of the sensitivity analysis highlight that the association between ED visits and group paediatric practice is stronger in the analysis where we included ED visits performed during daytime and week-days, while Model 3 where week-end or night non severe ED visits were included, the association between group CBP and ED visits was not significant; this indicates that the effect is present especially during primary care service working hours. Model 4, which considered the flu season which is critical for the ED workload, seems to confirm our hypothesis given that the OR is similar to model 2.

This is a population based study that used accurate information systems that allowed us to evaluate the effect of primary care in a country that has provided paediatric care free of charge since the 1970s. A major strength of the study is the fairly unique use of a large administrative data set to address the issue of non-urgent use of emergency services.

This study has some limitations. Even if parents are not informed of the type of practice model before registering their children with a CBP, we cannot exclude that family characteristics, such as anxiety, could have an effect either on CBP selection or on use the ED.

The use of information systems did not give us the opportunity to take into account the precise distance between the child's home and the CBP office or the nearest ED, or socioeconomic factors. Our study partially controlled for these confounders but we cannot exclude that additional confounding was present, moreover, patients exempt from payment could owe that status to the presence of a chronic disease, and not socio-economic difficulties. Another limitation is that our study did not permit to take into account of the organizational factors of different types of practice.

The generalizability of this study is found in several aspects of this analysis. First of all, it describes a method to evaluate a health care system and this method can be applied to different health care models. To date few studies have used administrative information systems to evaluate ED use for different primary care models. Secondly, in each health care system the attempt is to develop, especially for children with special health care needs, a primary care system able to be proactive, and able to ensure planned and coordinated care. In Italy, network and group paediatric care models were promoted, with financial incentives, to respond to these needs. This evaluation is part of the debate on quality and efficiency of different health care models.

## Conclusions

This study shows that the 14 per cent of children less than six years of age has been visited in Emergency Department for non urgent conditions. It shows also that there is an association between type of paediatrician primary practice and the use of the ED. Our results highlight the necessity of guaranteeing stronger availability of CBPs.

From a research perspective, there is a need for an in-depth investigation of parental attitudes on emergency service and primary care service to better investigate organizational factors associated with paediatric practice.

## Competing interests

The authors declare that they have no competing interests.

## Authors' contributions

SF participated in the design of the study and wrote the manuscript and helped to the statistical analysis. AP performed the statistical analysis, participated in the design of the study. FF, participated in the design of the study and helped to the statistical analysis. DDL conceived of the study, and participated in its design and coordination and helped to draft the manuscript. GG participated in the design of the study and coordination. All authors read and approved the final manuscript.

## Pre-publication history

The pre-publication history for this paper can be accessed here:

http://www.biomedcentral.com/1471-2296/11/32/prepub
